# Evaluation of autosomal dominant retinal dystrophy genes in an unaffected cohort suggests rare or private missense variants may often be benign

**Published:** 2013-05-06

**Authors:** Samuel P. Strom, Michael B. Gorin

**Affiliations:** Jules Stein Eye Institute, University of California Los Angeles, CA

## Abstract

**Background:**

Many genes have been reported as harboring autosomal dominant mutations causing retinal dystrophy. As newly available gene panel sequencing and whole exome sequencing will open these genes up to greater scrutiny, we assess the rate of rare coding variation in these genes among unaffected individuals to provide context for variants that will be discovered when clinical subjects are sequenced.

**Methods:**

Publicly available data from the Exome Variant Project were analyzed, focusing on 36 genes known to harbor mutations causing autosomal dominant macular dystrophy.

**Results:**

Rates of rare (minor allele frequency ≤0.1%) and private missense variants within autosomal dominant retinal dystrophy genes were found to occur at a high frequency in unaffected individuals, while nonsense variants were not.

**Conclusions:**

We conclude that rare missense variations in most of these genes identified in individuals with retinal dystrophy cannot be confidently classified as disease-causing in the absence of additional information such as linkage or functional validation.

## Introduction

Inherited retinal dystrophies (IRDs) can be collectively defined as a group of genetic disorders characterized by the progressive loss of photoreceptor function leading to impaired vision early in life. The major clinical diagnoses readily considered IRDs are Stargardt disease (STGD; OMIM #248200), retinitis pigmentosa (RP), cone/rod dystrophies (CRD), vitelliform macular dystrophy (VMD or Best disease; OMIM #153700), and occult macular dystrophy (OMD; OMIM #613587). As a group, these various forms of IRD occur in fewer than six individuals per 10,000, yet present a significant diagnostic burden to ophthalmic genetics due to phenotypic overlap and genetic heterogeneity. Clinical analysis, even when thorough and rigorously pursued, is often insufficient to connect a patient to a genetic defect. Many patients with IRDs present as sporadic cases without definitive family history of visual impairment, precluding the assessment of mode of inheritance.

Expanded mutation screening with multigene sequencing has been developed by several groups to address these diagnostic challenges. Various next-generation sequencing (NGS) techniques have been applied to screen for genetic variants causing retinal dystrophy, including research-based gene panels [1], diagnostic gene panels (Casey Eye Institute at Oregon Health and Science University, Portland, OR; Baylor College of Medicine, Houston, TX) and whole exome sequencing (WES) [2]. Early success with these technologies in providing otherwise cumbersome to obtain molecular diagnoses has fueled optimism in clinical molecular genetics, though not without trepidation [3,4].

Caution must be taken to avoid over-interpretation of results. Both laboratories and clinicians may be biased towards to providing meaningful results upon conducting exhaustive molecular testing, creating a potential conflict of interest in interpretation. It is our opinion that there is thus potential for over-assignment of causality to private heterozygous variants in genes associated with dominant disease.

The complete penetrance autosomal dominant mode of inheritance is often challenged by a lack of parental genetic material, asymptomatic carrier relatives, and other mitigating factors. This uncertainty is evident at the single gene level, but has become a major hurdle for multigene sequencing panels [5] and can compromise whole genome or whole exome testing if not properly addressed. Analysis of the phenotype most exhaustively tested with NGS, cardiomyopathy, has cast doubt on the pathogenicity of many variants reported to cause severe monogenic disease [6]. In this study, we use a similar approach to interrogate retinal dystrophies.

As of this writing, the Retinal Information Network (RetNet) [7], a manually curated online database of retinal disease genes, lists 36 genes responsible for autosomal dominant retinal dystrophy (adRD). Although identification of a novel or extremely rare variant in a patient with an IRD in one of these genes should be evaluated as a putative disease-causing variant, the clinical validity of such a causative relationship can be difficult to assess. Here we examine the spectrum of single nucleotide variants (SNVs) in these 36 genes in approximately 6,500 population controls using publicly available data in an attempt to put these findings into a broader context.

## Methods

### Data manipulation and statistical analysis

All data manipulations and statistical analyses were performed using custom notebooks within the Mathematica 9.0 computing environment (Wolfram Inc., Champaign, IL).

### Exome sequencing project data

The National Heart Lung and Blood Institute (NHLBI) Exome Sequencing Project (ESP) was designed to perform WES on a large cohort of individuals carefully phenotyped for several traits, particularly those related to cardiovascular health [8]. Retinal degeneration is not a trait selected for or against in any of the studies contained in the ESP. In aggregate, the project represents the largest publicly available control cohort for assessing mutational burden across most known genes.

Mean read depth (or “coverage”) data were downloaded from the Exome Variant Server [8] and filtered for all coding loci in 36 manually selected genes (approximately 32.6 kb). The percentage of loci sampled by at least 20 independent sequence reads on average was calculated for each gene (Table 1) to compare coverage between genes.

**Table 1:** Coding length, coverage, and variant summaries for 36 autosomal dominant retinal dystrophy genes culled from the EVS6500 database.

Gene	CDS	% Covered	Rare Variants		Private Variants	
			%	%	%	%
	Length	≥20x	Missense	Nonsense	Missense	Nonsense
*AIPL1*	1,152	100	0.43	0.05	0.29	0.03
*BEST1*	1,812	91.4	0.69	0.02	0.45	0.02
*CA4*	936	**53.8**	0.29	0	0.18	0
*CRB1*	**4,218**	82.7	**1.4**	0.05	0.91	0.05
*CRX*	897	100	0.31	0	0.2	0
*EFEMP1*	1,479	100	0.4	0	0.31	0
*ELOVL4*	942	100	0.22	0	0.18	0
*FSCN2*	1,548	100	0.75	0.02	0.51	0.02
*GUCA1A*	603	**33.3**	0.17	0	0.05	0
*GUCA1B*	600	94.8	0.26	0.05	0.18	0.03
*GUCY2D*	3,309	100	**1**	0	0.74	0
*HMCN1*	16,905	**73.4**	**6.19**	0.11	**4.14**	0.09
*IMPDH1*	1,797	100	0.46	0.03	0.26	0.03
*KLHL7*	1,758	**79.7**	0.26	0	0.23	0
*C1QTNF5*	1,737	100	0.86	0.02	0.6	0.02
*NR2E3*	1,230	**62.8**	0.49	0.02	0.31	0.02
*NRL*	711	**53.5**	0.18	0	0.09	0
*PITPNM3*	2,922	90.7	0.86	0	0.63	0
*PROM1*	2,595	96.9	**1**	0.05	0.65	0.03
*PRPF3*	2,049	100	0.23	0	0.22	0
*PRPF31*	1,497	93	0.32	0	0.25	0
*PRPF6*	2,823	99	0.35	0	0.28	0
*PRPF8*	7,005	99.9	0.4	0.02	0.32	0.02
*PRPH2*	1,038	100	0.22	0	0.14	0
*RHO*	1,044	100	0.49	0.02	0.31	0.02
*RIMS1*	**5,076**	88.5	**1.03**	0.02	0.78	0
*ROM1*	1,053	97	0.48	0.02	0.22	0.02
*RP1*	**6,468**	100	**1.91**	0.06	**1.34**	0.06
*RP1L1*	**7,200**	89.9	**4.17**	0.11	**2.77**	0.09
*RP9*	663	**77.7**	0.12	0	0.08	0
*RPE65*	1,599	100	0.45	0.03	0.34	0.03
*SEMA4A*	2,283	96.6	0.63	0	0.42	0
*SNRNP200*	**6,408**	99.8	0.85	0.02	0.65	0.02
*TIMP3*	633	81.2	0.06	0	0.02	0
*TOPORS*	3,135	98.2	0.82	0	0.6	0
*UNC119*	720	**71.8**	0.23	0.02	0.18	0.02

Rare is defined as having a minor allele frequency of <0.1%, including private variants (observed in only one individual). Boxes containing very long genes (95th percentile), coverage rates of <80%, and variant rates >1% are marked with bold text. These data suggest that when performing DNA sequencing of the complete coding sequence of the highlighted genes, rare missense variants are likely to be identified regardless of disease state. For such variants, their impact on disease will thus be difficult to interpret. Abbreviations: CDS, coding sequence; kb, kilobases; 20x, percentage of coding bases covered on average by at least 20 independent sequence reads.

SNV data from nearly 6,500 unrelated individuals is now publicly available through the Exome Variant Server [8], providing insights into genetic variants across the genome not previously available. Using Mathematica 9.0, we filtered out all SNVs to include only those predicted to alter an amino acid sequence (missense or nonsense) and present in ≤0.1% of individuals across 18,822 unique genes. Presence or absence from dbSNP was not used as an inclusion criterion.

To perform functional prediction of variants, we used the Condel analysis web site [9] by uploading a representative sample of the missense variants and downloading the results. We then compared the proportion of variants deemed “neutral” within all genes to that of a random sample of variants within the 36 autosomal dominant IRD genes from RetNet) [7].

Important to note, the nature of the data prevents the assignment of variants to specific individuals. As an example, this means that it is not possible to discern whether two unique SNV loci originally derive from two individuals or one. Therefore, though we must operate under the assumption that each variant represents a unique individual, we acknowledge will this will be incorrect in some cases. This may cause our analysis to over-estimate the SNV rate across individuals to some degree.

Also potentially problematic is the technical uncertainty of whole exome sequencing data. The methodologies implemented to perform variant discovery in the ESP have been previously validated as 95-99% concordant with Sanger sequencing or microarray genotyping [10]. While cryptic pseudogenes or unmapped gene family members and sequencing errors can generate false results, it does not appear that these issues confound general trends. For example, broadly applying an extremely conservative 10% false-positive rate to the ESP data would not significantly alter the overall interpretation of the results of this study.

## Results

### Coverage

The mean coverage of targeted bases across the entire genome in the EVS6500 [8] data is 81×. For each gene, the mean coverage of the coding bases across all samples was calculated (Table 1). While the majority of the genes are well covered, eight genes have <80% of the coding bases covered by 20 or more reads, including several genes with low coverage rates (<60%), carbonic anhydrase 4 (*CA4*), guanylate cyclase activator protein 1A (*GUCA1A*), and neural retina leucine zipper (*NRL*).

### Missense variants

A total of ~1.4 million rare missense SNVs were identified across all individuals and all genes. This translates to 216 such variants per person on average or one per ~51,000 coding bases. Private variants account for only 17,597 (1.3%) of rare missense SNVs. Thus individuals carry on average 2.7 private missense variants.

The 36 adRD genes comprise 0.29% (98kb out of 33 Mb) of the total coding sequence length of all genes analyzed. Thus the expected rates of rare and private missense variants are approximately 0.64 and 1/125 respectively. As observed values of 0.71 and 1/94 respectively do not significantly deviate from expected values, we may consider these genes to be typical in this respect.

**Figure 1:**
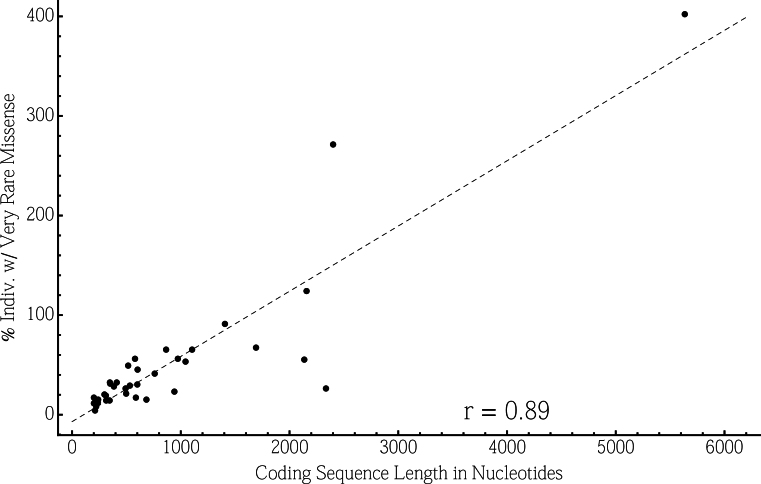
Rare Variant Identification is Correlated With Coding Sequence Length. Coding sequence (CDS) length in nucleotides is plotted against the proportion of individuals carrying a very rare (MAF <0.1%) missense variant. A very strong positive correlation is observed (Pearson’s correlation coefficient r = 0.89), indicating that genes with long coding sequences are more likely to have a high rate of rare missense variants independent of the functional impact of those variants.

Of the 36 adRD genes analyzed, seven have a rare missense variant in ≥1% of putatively unaffected individuals (Table 1). Particularly troubling are *HMCN1* (hemicentin 1) and *RP1L1* (retinitis pigmentosa 1-like 1), both with rare missense rates of >4%. Neither gene is uncommonly susceptible to benign missense variants; rather, these genes simply have long coding sequences. These genes, as well as the four other genes with a coding sequence length marked in bold in Table 1, are in the 95^th^ percentile of the longest coding genes in the human genome. As dictated by random sampling precepts, CDS (coding sequence) length is strongly correlated with rare missense variant rates across the 36 adRD genes (Figure 1, Pearson’s correlation coefficient=0.89).

Even when restricted to private variants, SNVs observed in only a single individual, these genes still have alarmingly high rates of heterozygous missense variants. Using an extremely conservative estimate (setting the average number of individuals per SNV at 0.5), at least 10% of the individuals studied carry such a variant. Only two genes, *TIMP3* 3 (tissue inhibitor of metalloproteinases) and *RP9* (retinitis pigmentosa 9 ), have adequate coverage (81% and 78%, respectively) and a private missense variant rate <0.1%. Both genes have short coding sequences (633 and 663 nucleotides, respectively) and are thus small targets for random mutation.

Functional prediction algorithms such as PolyPhen [11] and SIFT [12] are often referred to when attempting to assess the correlation between missense variants and disease. We implemented Condel, a method combining three prediction algorithms (PolyPhen [11] and SIFT [12], and Mutation Assessor [13]), on 1,626 missense variants within these genes. On average, 29% of these variants are predicted to be benign. This value is consistent with an exome study of intellectual disability [14]. Even with this 29% neutral variant rate applied across all missense SNVs studied, 24 of the 36 genes still harbor a potentially deleterious missense rate >0.1% or 1:1,000, exceeding even the most liberal estimates of the prevalence of adRD. From this analysis, we must conclude that the majority of heterozygous missense variants detected with NGS in these 36 adRD genes do not cause retinal dystrophy with high penetrance.

### Nonsense variants

For stochastic reasons, SNVs introducing a premature stop codon are expected to be less common than synonymous and non-synonymous missense variants. The EVS data support this logic, as only 44 rare nonsense mutations are present. Most genes had three or fewer, equivalent to a rate of <0.05% (Table 1). These variants may represent sequencing errors in some cases, but this low rate is consistent with our expectations for partially penetrant alleles, or true disease-causing mutations in affected individuals not screened out by the NHLBI. There was little difference between rare and private nonsense SNVs. Due to the scarcity of nonsense SNVs in the putatively unaffected public, we conclude that the identification of such a variant in any of these genes in a sporadic IRD proband is highly suggestive of causality.

## Discussion

Gene sequencing panels and genome-wide mutation screening represent the leading edge of clinical molecular diagnostics [1,2]. These techniques can dramatically reduce the time, cost, and energy spent identifying a molecular diagnosis for heritable conditions. Individuals with IRD are primed to benefit greatly from these advances due to the high level of phenotypic, locus, and allelic heterogeneity.

Public resources such as the ESP analyzed in this study provide valuable, if indirect, clues into the pathogenicity of variant classes within known disease genes. Although NGS techniques are subject to specific types of errors (incomplete coverage, mismapping, random sampling errors, etc.), general trends can be observed when looking at large data sets. In this study, we use this public resource to interrogate autosomal dominant retinal dystrophy genes. Similar approaches have yielded valuable results for cardiomyopathy genes [10]. Validation of these concepts with a large clinical cohort sequenced by WES is necessary and will be pursued in future studies.

A major reason that some genes are found to have relatively high variant rates is simply that the genes are longer (Figure 1). As this concept is often overlooked in gene discovery efforts, false-positive gene associations can easily occur with sporadic cases or small pedigrees.

While linkage studies taking advantage of large pedigrees have successfully identified these genes as harboring likely disease-causing mutations, such evidence cannot be applied to a new proband in a vacuum. Our analysis of typical human exomes suggests that rare or private missense variants within adRD genes are, in aggregate, a common occurrence.

Prior to utilizing WES for clinical or research purposes, it is critical to assess whether the genes most relevant to a disease phenotype are thoroughly examined. Several genes, including *CA4*, *GUCA1A*, and *NRL* are poorly covered by the experiments studied here. As laboratories may differ in coverage of specific genes, this information should be made available to researchers and clinicians and considered prior to ordering WES testing. This limitation should be weighed when comparing WES with disease-specific gene panels, which tend to provide more reliable coverage among established genes but are not flexible enough to sample recently identified genes and cannot identify novel disease genes.

Detailed genotype-phenotype analysis, such as the relationship between *RP1L1* variants and occult macular dystrophy [15], demonstrates that genes with apparent dominant inheritance often have a complex relationship with disease. This complexity is typically not adequately conveyed by large databases and can provide a time-consuming source of false-positive results.

Much of the ambiguity of the contribution of missense variants to dominant disease can be resolved when family history and family genetic materials are available. Many true causal mutations have been identified as either segregating in large pedigrees or arising de novo in a proband. As establishing a variant as de novo requires genotyping both parents, in the context of WES, analyzing parent-offspring trios from the outset is extremely powerful because typical individuals carry only two or fewer de novo coding variants [14,16].

Familial samples, usually comprised of trios including the affected individual and parents or other informative relatives, are invaluable for determining when variants in the same gene are in *cis* or *trans* configurations. Unfortunately, familial samples are often unavailable for genetic analysis, particularly in late-onset diseases. In such cases, pretest counseling about the likelihood of success of gene panel or WES testing should clarify that these tests are susceptible to the identification of variants of uncertain significance (VUS).

Small insertions and deletions (indels) are either inherited or sporadic variants in which a sample genome differs from the reference genome by a net gain or loss of 1–50 bp. Short read parallel sequencing can reliably detect small indels, typically up to 10–20 bp. This class of variant, when present in the open reading frame of a gene, can cause a gain or loss of amino acids (if a multiple of three) or a frameshift. Unfortunately, due to sequence depth constraints, no databases such as the EVS provide sufficient indel information among a control population to assess the rates of these two classes of coding indels. However, it is safe to assume that frameshift indels function akin to nonsense SNVs and are highly likely to reduce eventual protein expression of that allele. In-frame indels are more difficult to assess, and may require specific knowledge of key amino acids and functional domains to properly interpret.

SNVs within the immediate two bases surrounding an exon boundary are documented within the EVS as splice site variants. We chose not to include these variants in this analysis, as these variants are difficult to classify as missense or nonsense (roughly one third of exons putatively skipped would be in-frame) and too rare to provide meaningful information as a separate group. Although variants at canonical splice donor and acceptor sites reliably alter splicing, the function effect of this change cannot be predicted with certainty. Instances in which the abolition of a splice donor or acceptor site leads to a predicted frameshift in the resulting mRNA are functionally identical to nonsense SNVs in most cases; however, in-frame splice alternates may or may not impair the function of the downstream gene product. Potential splice variants must thus be carefully examined before a functional prediction is assigned.

Unlike allele specific genotyping, gene sequencing introduces the complicating factor of VUS. Sporadic cases of IRD are particularly susceptible to uncertainty, as many different genes must be included on gene panels and in directed analysis of WES. Our study of variation among an unaffected population suggests that, while nonsense variants in adRD genes are extremely uncommon and highly likely to be pathologic, missense variants are far more uncertain in terms of being designated as disease-causing. Undoubtedly, some rare or private missense variants can be causative or partially penetrant. However, observation of such a variant in a single patient with IRD is not sufficient information to assign causality. Caution and skepticism are important in such cases, despite the understandable desire of clinicians and patients to come to a definitive conclusion.
